# Differential ability of the dorsal and ventral rat hippocampus to exhibit group I metabotropic glutamate receptor–dependent synaptic and intrinsic plasticity

**DOI:** 10.1177/2398212816689792

**Published:** 2017-01-24

**Authors:** Patrick Tidball, Hannah V. Burn, Kai Lun Teh, Arturas Volianskis, Graham L. Collingridge, Stephen M. Fitzjohn

**Affiliations:** 1 School of Physiology, Pharmacology and Neuroscience, University of Bristol, Bristol, UK; 2 Department of Physiology, University of Toronto, Toronto, Ontario, Canada; 3 Lunenfeld-Tanenbaum Research Institute, Mount Sinai Hospital, Toronto, Ontario, Canada

**Keywords:** Long-term depression, EPSP-to-spike coupling, metabotropic, metabotropic glutamate receptor, (*RS*)-3,5-dihydroxyphenylglycine

## Abstract

**Background:**

The hippocampus is critically involved in learning and memory processes. Although once considered a relatively homogenous structure, it is now clear that the hippocampus can be divided along its longitudinal axis into functionally distinct domains, responsible for the encoding of different types of memory or behaviour. Although differences in extrinsic connectivity are likely to contribute to this functional differentiation, emerging evidence now suggests that cellular and molecular differences at the level of local hippocampal circuits may also play a role.

**Methods:**

In this study, we have used extracellular field potential recordings to compare basal input/output function and group I metabotropic glutamate receptor-dependent forms of synaptic and intrinsic plasticity in area CA1 of slices taken from the dorsal and ventral sectors of the adult rat hippocampus.

**Results:**

Using two extracellular electrodes to simultaneously record field EPSPs and population spikes, we show that dorsal and ventral hippocampal slices differ in their basal levels of excitatory synaptic transmission, paired-pulse facilitation, and EPSP-to-Spike coupling. Furthermore, we show that slices taken from the ventral hippocampus have a greater ability than their dorsal counterparts to exhibit long-term depression of synaptic transmission and EPSP-to-Spike potentiation induced by transient application of the group I mGluR agonist (*RS*)-3,5-dihydroxyphenylglycine.

**Conclusions:**

Together, our results provide further evidence that the information processing properties of local hippocampal circuits differ in the dorsal and ventral hippocampal sectors, and that these differences may in turn contribute to the functional differentiation that exists along the hippocampal longitudinal axis.

## Introduction

The hippocampus is a forebrain structure that plays critical roles in memory formation and anxiety-related behaviour (Bannerman et al., 2014; Squire et al., 2004). In rodents, the hippocampus forms a distinctive ‘C’ shape, with a longitudinal axis running from the septal pole rostrodorsally to the temporal pole caudoventrally. Although the rodent hippocampus is relatively uniform along this longitudinal axis in terms of its intrinsic connectivity and cytoarchitecture (Amaral and Lavenex, 2007; Andersen et al., 1971a), there is now strong evidence to suggest that it can be divided into at least three functionally distinct domains: dorsal (DH), intermediate (IH) and ventral (VH) (Fanselow and Dong, 2010; Moser and Moser, 1998; Strange et al., 2014). In particular, lesion studies have shown that spatial learning is largely a function of the DH, whereas the VH is involved in the processing of information with emotional or motivational salience (Bannerman et al., 2003; Fanselow and Dong, 2010; Moser et al., 1993). Although it is likely that this functional differentiation can be explained, at least in part, by differences in extrinsic connectivity (Strange et al., 2014; Swanson and Cowan, 1977), emerging reports suggest that differences at the local circuit level may also play a role. For example, the DH and VH have been shown to differ in their relative expression levels of ionotropic glutamate receptor subunits (Pandis et al., 2006), in the intrinsic properties and morphological characteristics of their principal cells (Dougherty et al., 2012), as well as in their basal synaptic efficacy and capacity to exhibit synaptic long-term potentiation (LTP) (Maggio and Segal, 2007a; Papatheodoropoulos and Kostopoulos, 2000a; Pofantis and Papatheodoropoulos, 2014).

The ability of synapses to undergo long-lasting changes in efficacy is thought to underlie learning and memory processes (Bliss and Collingridge, 1993). In addition to LTP, two major forms of synaptic long-term depression (LTD) coexist at hippocampal CA1 synapses: one dependent on the activation of *N*-methyl-d-aspartate (NMDA) receptors (NMDAR-LTD; Collingridge et al., 2010; Dudek and Bear, 1992) and the other on the activation of group I metabotropic glutamate receptors (mGluRs, comprising mGlu1 and mGlu5; mGluR-LTD; Fitzjohn et al., 1999; Huber et al., 2000; Palmer et al., 1997). In contrast to NMDAR-LTD, which is more pronounced early in development (Massey et al., 2004; Yang et al., 2005), mGluR-LTD can be readily induced in slices from older animals by either electrical or pharmacological stimulation (Kumar and Foster, 2007; Moult et al., 2008), and it may therefore represent the dominant form of LTD in the adult hippocampus. Moreover, long-lasting changes in intrinsic neuronal excitability induced by group I mGluR activation may also play a role in information processing in mature hippocampal circuits (Clement et al., 2009; Ireland et al., 2004; Piccinin et al., 2008). Interestingly, group I mGluR-dependent processes have been implicated in behavioural paradigms typically associated with segregated hippocampal function, such as spatial learning (Ayala et al., 2009; Balschun et al., 1999) and fear conditioning (Bast et al., 2001; Riedel et al., 2002; Schulz et al., 2001; Wang et al., 2013). However, surprisingly little is known about mGluR signalling in the DH versus the VH, and whether or not the DH and VH differ in their ability to exhibit mGluR-dependent plasticity has not previously been examined.

In this study, we have investigated differences in group I mGluR-mediated plasticity along the longitudinal axis of the hippocampus. Using electrophysiological recordings from area CA1 of adult rat hippocampal slices prepared in the sagittal (DH) and horizontal (VH) orientations, we found that a brief bath application of the selective group I mGluR agonist (*RS*)-3,5-dihydroxyphenylglycine (DHPG) resulted in substantially larger magnitudes of synaptic LTD and EPSP-to-spike (E-S) potentiation in VH compared to DH slices. The use of an alternative slice preparation method to obtain transverse DH and VH slices confirmed that this difference in the magnitude of DHPG-LTD was due to the dorsal versus ventral origin of the slices rather than the angle of cutting. We also found the enhanced DHPG-LTD in VH slices to be associated with a larger increase in paired-pulse facilitation (PPF) when compared to the LTD induced in DH slices, suggesting a greater contribution of a presynaptic expression mechanism to this form of plasticity in the VH. Furthermore, we describe a long-lasting depression of the maximal population spike (PS) that occurs after DHPG treatment, which is predominantly apparent in slices from the VH. Our results suggest that the VH has a greater overall ability to exhibit group I mGluR-dependent forms of plasticity when compared to the DH.

## Materials and methods

### Animals

Adult male Wistar rats (7–10 weeks old) were obtained from Charles River Laboratories (Margate, UK) and kept in standard housing conditions with controlled temperature (21°C ± 2°C) and a 14/10 h light/dark cycle (lights on at 5.00 a.m./off at 7.00 p.m.). Animals were housed in groups of four per cage and food and water were available ad libitum. Following transport and delivery, animals were allowed to settle for a minimum of 1 week prior to use in experiments. All experiments were conducted in accordance with the UK Animals (Scientific Procedures) Act.

### Hippocampal slice preparation

**Figure 1.**
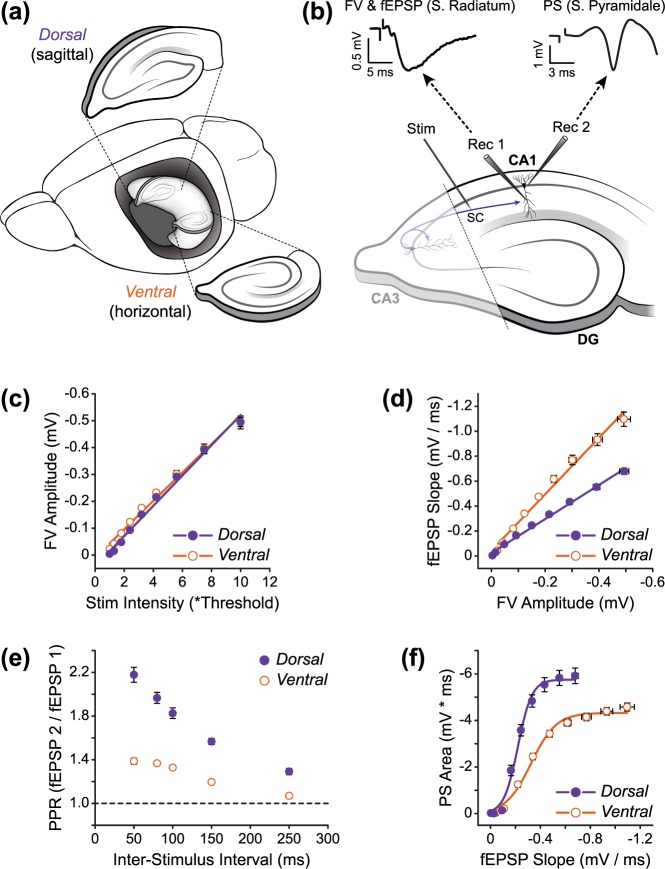
Synaptic transmission and E-S coupling differ between dorsal and ventral hippocampal slices. (a) Schematic showing the origin of dorsal (DH) and ventral (VH) hippocampal slices. (b) Schematic showing electrode placement in a hippocampal slice. Recording electrode 1 (Rec 1) in the stratum radiatum records the fibre volley (FV) and fEPSP, whereas Rec 2 placed in the stratum pyramidale records the population spike (PS). Responses are evoked by an electrode (Stim) placed at the CA2/CA1 border, stimulating Schaffer collateral fibres (SC). (c–f) Electrophysiological characterisation of DH and VH slices. (c) No difference was observed in the relationship between stimulus intensity (plotted as multiples of the threshold intensity for evoking a fEPSP) and the FV. However, basal transmission was reduced in DH versus VH slices (d), which correlated with a larger PPF in DH (e). (f) In contrast, E-S coupling was enhanced in DH slices relative to VH slices. For the I/O curve data in panels (c), (d) and (f), *n* = 24 and 28 for DH and VH, respectively. For the PPF data in panel (e), *n* = 5 for both DH and VH. In this and subsequent figures, filled symbols represent data from DH, whereas open symbols represent data from VH. Each point represents the mean ± SEM.

**Figure 2.**
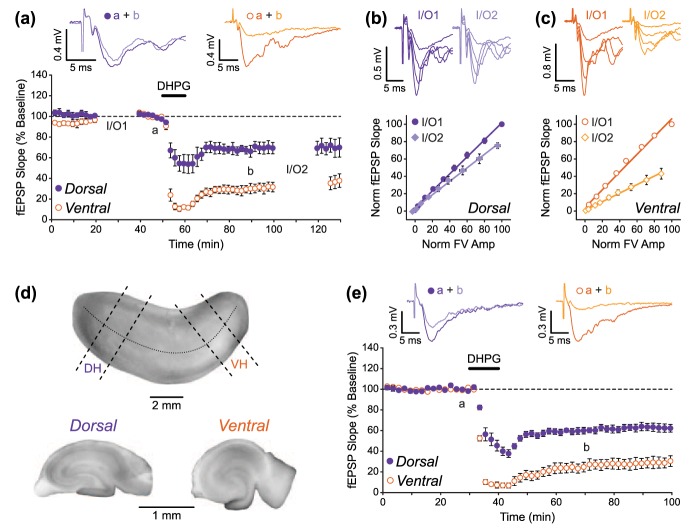
mGluR-LTD of fEPSPs is larger in ventral than in dorsal hippocampal slices. (a) Pooled data (*n* = 5 and 5) showing that LTD induced by application of the group I mGluR agonist DHPG (100 µM) is greater in VH than in DH slices. Example traces from the time points indicated (a and b) are shown above. DHPG was applied for the time indicated by the bar. Gaps in recording are due to construction of input/output (I/O) curves. (b and c) The FV-fEPSP relationship was reduced by DHPG application in both (b) DH and (c) VH, but to a greater extent in VH. Data are normalised to the baseline maximum FV amplitude and fEPSP slope measurement in individual experiments. Example traces for I/O curves at the two time points are shown above. (d and e) The difference in the magnitude of mGluR-LTD was still seen when experiments were performed using transverse DH and VH slices.

On the day of experiment, individual animals were briefly transported from the in-house animal holding facility to a preparation room within the same building where they were deeply anaesthetised by isoflurane inhalation and killed by cervical dislocation. Rats were then decapitated and their brains were rapidly removed and placed in ice-cold cutting saline composed of (in mM) 87 NaCl, 2.5 KCl, 25 NaHCO_3_, 1.25 NaH_2_PO_4_, 25 d-glucose, 0.6 ascorbic acid, 75 sucrose, 7 MgCl_2_, and 0.5 CaCl_2_ (bubbled with 95% O_2_–5% CO_2_). After being allowed to cool for 2–3 min, brains were transferred onto filter paper saturated with ice-cold cutting saline. The cerebellum and a small section of frontal cortex were removed by scalpel cut, and brains were hemisected along the longitudinal fissure. Flat surfaces for mounting each hemisphere were created by making blocking cuts on either the lateral surfaces parallel to the sagittal plane (for DH slices) or on the dorsal surfaces parallel to the horizontal plane (for VH slices). The hemispheres were then mounted onto the stage of a DTK-1000 vibrating microtome (DSK, Kyoto, Japan), and sagittal or horizontal sections (400 µm thick) were cut in ice-cold cutting saline to obtain DH or VH slices, respectively (a schematic diagram showing the origin of DH and VH slices prepared using this method is shown in Figure 1(a)). In one set of experiments (Figure 2(d) and (e)), transverse hippocampal slices from the DH or VH sectors were prepared using an alternative method. For these experiments, whole hippocampi were first isolated from the hemisected brains before being carefully adhered to agar blocks on their flat surfaces (the dentate gyrus) by wicking away excess cutting saline using filter paper. Scalpel cuts, perpendicular to the hippocampal longitudinal axis, were made through the hippocampi and agar in order to create flat surfaces for mounting, leaving either the dorsal or ventral two-thirds of the hippocampus intact depending on the type of slices (dorsal or ventral) being prepared. The hippocampi, supported by the agar blocks, were then mounted vertically onto the stage of the vibrating microtome with their dorsal or ventral tips pointing upwards and their longitudinal axis perpendicular to the microtome blade. DH or VH transverse slices (400 µm thick) were then cut in ice-cold cutting saline. For both slice preparation methods, a maximum of four DH or VH slices (starting with the first slice in which all the hippocampal sub-regions were clearly visible) were kept for experiments to ensure a clear separation between the DH and VH sectors. The CA3 region was removed immediately after sectioning in all cases and slices were then transferred to a holding chamber containing artificial cerebrospinal fluid (ACSF) composed of (in mM) 124 NaCl, 3 KCl, 26 NaHCO_3_, 1.4 NaH_2_PO_4_, 10 d-glucose, 1 MgSO_4_, and 2 CaCl_2_ (bubbled with 95% O_2_–5% CO_2_). Slices were allowed to recover at room temperature for a minimum of 1 h before use.

### Electrophysiology

After being allowed to recover, DH or VH slices were transferred to a submerged recording chamber, maintained at 31°C, and perfused with oxygenated ACSF at a rate of 2 mL/min. For all experiments, the NMDA receptor antagonist L-689,560 (5 µM) and the GABA_A_ receptor blocker picrotoxin (50 µM) were added to the ACSF and were continuously present. Extracellular field potentials were recorded from area CA1 using glass microelectrodes (2–3 MΩ) filled with ACSF. Two recording electrodes were used to simultaneously record (1) fibre volleys (FVs) and field EPSPs (fEPSPs) from the stratum radiatum and (2) PSs from the stratum pyramidale. Responses were evoked by stimulation of the Schaffer collaterals using a platinum–iridium bipolar electrode (FHC, Bowdoin, ME, USA) placed in the stratum radiatum at the CA1–CA2 border, and stimuli (100 µs duration) were delivered once every 30 s via a DS3 constant current stimulus isolator (Digitimer Ltd, Welwyn Garden City, UK). A schematic diagram showing the positions of the stimulating and recording electrodes is shown in Figure 1(b). After placement of the electrodes, responses were allowed to stabilise for a minimum of 20 min and stimulus intensity was then set at two to three times the fEPSP threshold in order to evoke submaximal fEPSPs and PSs for baseline recording. Responses were amplified using a Multiclamp 700B amplifier (Molecular Devices, Sunnyvale, CA, USA) and were digitised at 40 kHz, monitored online, and recorded to a personal computer using WinLTP software (Anderson and Collingridge, 2007). For offline analysis, responses were low-pass filtered and measured as averages of four consecutive sweeps. FVs and fEPSPs were filtered at 2 kHz and quantified by their peak amplitude and initial slope, respectively, using WinLTP software. PSs were filtered at 1 kHz and quantified by their area using Platin Calculator (custom software courtesy of Morten S. Jensen, Aarhus University, Denmark).

### Input/output curves

Input/output (I/O) curves were generated during periods of stable baseline recording and 40 min after DHPG treatment, as indicated in the results. Data for the I/O curves were obtained by varying the stimulation intensity over a range of nine pre-determined values. The lowest intensity was taken as the threshold for evoking a visually detectable fEPSP (defined as 1× intensity), and the eight remaining values were taken as multiples of this threshold, as follows: 1.3×, 1.8×, 2.4×, 3.2×, 4.2×, 5.6×, 7.5×, and 10×. This logarithmic scale was chosen (after some testing) so that the full range of the PS (from subthreshold to supramaximal) would be sampled in the majority of cases. Four sweeps were recorded at each stimulus intensity and averaged together for measurement of the resulting field potentials. I/O curves were analysed using the built-in functions in Graphpad Prism 5 software (Graphpad Software Inc., San Diego, CA, USA). The linear stimulus-to-FV (stim-FV) and FV-to-fEPSP (FV-EPSP) curves were fitted with a straight line and the slope values were used for quantification of the I/O relationships. E-S curves were fitted with a sigmoidal (variable slope) function, and the E_50_ value (defined as the size of the fEPSP at the half-maximal PS) was used to quantify E-S coupling. For within-experiment comparisons of I/O curves (before and after DHPG treatment), responses were normalised to their maximal values in baseline (defined as 100%) and percentage changes in slope, E_50_, or maximal PS were calculated as appropriate. For comparison of basal I/O curves between DH and VH slices, raw (non-normalised) data were used for curve fitting, and the resulting slope, E_50_, or maximal PS values were compared directly.

### PPF

**Figure 3.**
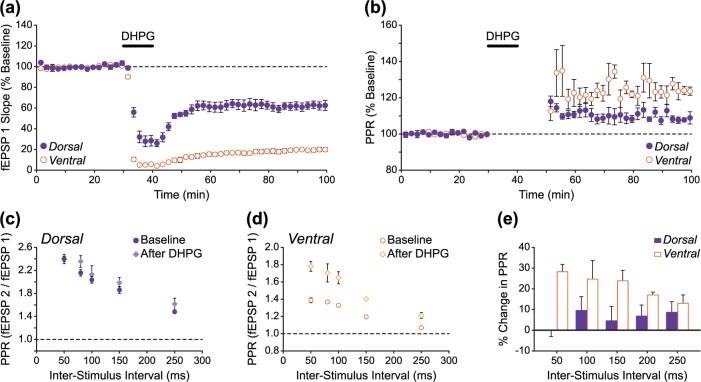
Increased paired-pulse facilitation following induction of DHPG-LTD is larger in ventral versus dorsal hippocampal slices. (a and b) Pooled fEPSP data showing experiments in which paired stimuli (100 ms inter-stimulus interval) were delivered every 30 s throughout the experimental time course in DH and VH slices (*n* = 5 and 5). Compared to DH slices, the larger magnitude DHPG-LTD in VH slices (a) was accompanied by a larger increase in PPF (b). Note that PPF could not be accurately measured during and immediately following DHPG application due to the large depression of fEPSPs during these periods, especially in VH slices. These data points have therefore been omitted in (b). (c–e) PPF measured across a range of inter-stimulus intervals 30 min before (baseline) and 1 h after DHPG treatment in (c) DH and (d) VH slices. Across the range of inter-stimulus intervals as a whole, the percentage increase in PPF after DHPG was significantly larger (*p* < 0.01, two-way ANOVA) in VH compared to DH slices (e).

PPF of fEPSPs was assessed by delivering paired stimuli every 30 s across a range of inter-stimulus intervals as follows (in ms): 50, 80, 100, 150, and 250. Four sweeps were recorded at each inter-stimulus interval and averaged together for measurement of the resulting field potentials. PPF was measured both under basal conditions (after stabilisation of responses and just prior to baseline recording) and 1 h after washout of DHPG. For measurement of PPF across an experimental time course (Figure 3(b)), a fixed inter-stimulus interval of 100 ms was used. The paired-pulse ratio (PPR) was calculated by dividing the slope of the second fEPSP by the slope of the first fEPSP.

### Drugs

(*RS*)-DHPG, picrotoxin, and L-689,560 were obtained from Abcam (Cambridge, UK). All drugs were prepared as stock solutions according to the supplier’s recommendations and stored at −20°C until use. On the day of experiment, drugs were diluted to their final concentration in ACSF and bath applied to the slices via the perfusion system. Picrotoxin and L-689,560 were present in the ACSF throughout all experiments, and DHPG was applied for 10 min as indicated in the figures.

### Data presentation and statistics

All pooled data are presented as mean ± standard error of mean (SEM). For each set of experiments, each *n* represents a slice from a different animal. Data in the time course plots are normalised to the pre-DHPG baseline, which is defined as 100% (for experiments with I/O curves, time course plots are normalised to the 10-min baseline period after I/O1 and before DHPG application). The magnitude of LTD in individual experiments was assessed as either the percent depression of fEPSPs from baseline in the period 30–40 min after the start of DHPG washout (for the experiments without I/O curves shown in Figure 2(e)) or as the percent change in the slope of the FV-EPSP curve for experiments in which I/O curves were generated before and 40 min after DHPG treatment. Statistical differences were assessed using Student’s *t*-test for paired or unpaired data, as appropriate. Comparison of PPF data between DH and VH slices across a range of inter-stimulus intervals was performed using two-way analysis of variance (ANOVA). Concentration–response curves were analysed using the built-in functions in Graphpad Prism 5 software. The ‘sigmoidal dose–response (variable slope)’ function was used for curve fitting, and statistical comparisons of the upper asymptotes and EC_50_ values were made using the extra sum-of-squares *F* test.

## Results

### The DH and VH CA1 differ in their basal levels of synaptic transmission and E-S coupling

Although the DH and VH share the same anatomical arrangement of principal cells and connections, they have been shown to differ in a number of ways at the molecular level and in the intrinsic properties of their individual neurons (Dougherty et al., 2012; Leonardo et al., 2006; Pandis et al., 2006). As such, subtle differences in synaptic connectivity and information processing may exist between the DH and VH sectors. Indeed, one report found that the same presynaptic activation resulted in a greater synaptic response in the CA1 region of VH compared to DH slices (Pofantis and Papatheodoropoulos, 2014), though it should be noted that other studies have found no difference in basal synaptic efficacy along the dorsoventral hippocampal axis (Maggio and Segal, 2007a, 2007b).

In this study, we prepared DH and VH slices by cutting along the sagittal and horizontal planes of the hemisected rat brain, respectively (see section ‘Materials and methods’; Figure 1(a)), and all experiments were performed in the continuous presence of the NMDA receptor antagonist L-689,560 (5 µM) and the GABA_A_ receptor blocker picrotoxin (50 µM). Basal I/O function in area CA1 was therefore examined under these experimental conditions by comparing I/O curves generated in ‘naïve’ DH (*n* = 24) and VH (*n* = 28) slices during periods of stable baseline recording. The use of two extracellular recording electrodes to simultaneously record FVs and fEPSPs from the stratum radiatum and PSs from the stratum pyramidale (Figure 1(b)) allowed us to compare three I/O relationships: (1) the level of presynaptic fibre activation in response to afferent stimulation, (2) synaptic efficacy, and (3) E-S coupling – a measure of CA1 pyramidal cell firing efficiency in response to excitatory input (Andersen et al., 1971b, 1980; Marder and Buonomano, 2003). In a separate set of experiments, we also compared basal levels of PPF in DH and VH slices (*n* = 5 for both) under the same set of recording conditions. PPF – a form of short-term plasticity – is widely considered to depend on presynaptic mechanisms for its expression and can be used as an indicator of the probability of transmitter release in a given population of synapses (Dobrunz and Stevens, 1997; Zucker and Regehr, 2002).

The relationship between the stimulus intensity and the afferent FV (Stim-FV) was the same in both DH and VH slices, indicating that a similar number of afferent fibres were activated for a given stimulus intensity in both slice preparations (Figure 1(c); line slopes: DH = −0.056 ± 0.002, VH = −0.053 ± 0.003; unpaired *t*-test: *t*_(50)_ = 1.05, *p* > 0.05). In contrast, we found basal synaptic transmission to be markedly different in DH and VH slices. In DH slices, the FV-to-fEPSP (FV-EPSP) curve had a slope value of 1.38 ± 0.06, whereas in VH slices the fitted slope was considerably steeper with a value of 2.30 ± 0.11, demonstrating that the strength of synaptic transmission was significantly greater in VH slices (Figure 1(d); unpaired *t*-test: *t*_(50)_ = 7.26, *p* < 0.01). Moreover, this enhanced excitatory transmission in the VH correlated with significantly lower levels of PPF in VH compared to DH slices across the range of inter-stimulus intervals tested, suggesting that a higher overall release probability at VH versus DH CA3-to-CA1 synapses may be largely responsible for the difference in synaptic efficacy (Figure 1(e); two-way ANOVA: *F*_(1,40)_ = 488.3, *p* < 0.01). The E-S curves also differed significantly between DH and VH slices (Figure 1(f)). In terms of E-S coupling, we found that the size of the fEPSP required to elicit a half-maximal PS was significantly smaller in DH slices compared to VH slices, indicating a stronger coupling of excitatory input to cell firing in the DH versus the VH (DH E_50_ = −0.22 ± 0.01 mV/ms, VH E_50_ = −0.32 ± 0.01 mV/ms; unpaired *t*-test: *t*_(50)_ = 6.74, *p* < 0.01). Furthermore, the maximal PS that could be elicited was found to be larger in DH slices, suggesting that a greater number of cells were able to contribute to the population response in slices from the DH (DH maximal PS = −5.78 ± 0.32 mV*ms, VH maximal PS = −4.33 ± 0.16 mV*ms; unpaired *t*-test: *t*_(50)_ = 4.24, *p* < 0.01). These results show that although the DH and VH CA1 have the same overall intrinsic anatomy, they differ in their synaptic and integrative properties, with the DH exhibiting a lower basal level of glutamatergic synaptic transmission compared to the VH, but a higher propensity for action potential discharge in response to excitatory input.

### DHPG-induced synaptic LTD is larger in the VH compared to the DH

mGluR-dependent LTD of synaptic transmission is often studied in its chemically induced form – by bath application of the selective group I mGluR agonist DHPG (Gladding et al., 2009; Schoepp et al., 1994). Here, we have investigated whether this DHPG-induced synaptic LTD (DHPG-LTD) differs in the dorsal and ventral regions of the hippocampus. In line with previous studies (Fitzjohn et al., 1999; Moult et al., 2002; Palmer et al., 1997), a brief application of (*RS*)-DHPG (100 µM, 10 min) resulted in an initial acute depression followed by LTD of the fEPSP in both DH and VH slices. Interestingly, however, we found that LTD was considerably larger in VH compared to DH slices (Figure 2(a); *n* = 5 for both). For quantitative analysis, we compared I/O curves generated before DHPG application and 40 min after DHPG washout in both DH and VH slices (denoted I/O1 and I/O2, respectively, in Figure 2(a)). In DH slices, LTD was measured as a −22% ± 4% change in the slope of the FV-EPSP curve (Figure 2(b)), whereas a −55% ± 8% change in the slope was measured in VH slices (Figure 2(c); unpaired *t*-test (DH vs VH): *t*_(8)_ = 3.85, *p* < 0.01).

Previous studies have shown that different methods of hippocampal slice preparation – and in particular slice orientation – can have an impact on experimental outcomes. For example, the mechanisms involved in the induction of LTP at mossy fibre-to-CA3 synapses have been reported to differ in sagittal versus transverse slices (Sherwood et al., 2012), and the precise involvement of the GluN2B receptor subunit in NMDAR-LTD at CA1 synapses depends on whether slices are prepared in a sagittal or coronal orientation (Bartlett et al., 2011). Therefore, to confirm that the difference in the magnitude of LTD in our experiments was a true reflection of the dorsal versus ventral origin of the slices, rather than some other consequence of our slicing procedure, we also examined DHPG-LTD in DH and VH slices prepared using an alternative method. In these experiments, slices were prepared by first dissecting the hippocampus out from its surrounding structures and then by slicing perpendicular to the longitudinal hippocampal axis to obtain transverse slices from either the DH or VH sectors (see section ‘Materials and methods’ for the detailed procedure; Figure 2(d)). In agreement with our previous findings, the same brief application of DHPG (100 µM, 10 min) resulted in a substantially larger magnitude of LTD in VH transverse slices compared to DH transverse slices (Figure 2(e); DH slices = 39% ± 3% LTD, VH slices = 73% ± 5% LTD; *n* = 6 for both datasets; unpaired *t*-test (DH vs VH): *t*_(10)_ = 6.12, *p* < 0.01).

### DHPG-LTD is associated with a larger change in PPF in VH compared to DH slices

The locus of mGluR-LTD expression is still a matter of debate, with some studies providing evidence for a presynaptic mechanism (Fitzjohn et al., 2001; Qian and Noebels, 2006; Tan et al., 2003; Watabe et al., 2002), and others advocating for a postsynaptic expression (Moult et al., 2006; Waung et al., 2008; Xiao et al., 2001; Zhang et al., 2008). Based on previous reports that mGluR-LTD is accompanied by an increase in PPF (Faas et al., 2002; Fitzjohn et al., 2001; Kumar and Foster, 2007; Tan et al., 2003), and because we found basal transmission and PPF to be markedly different in naïve DH and VH slices (Figure 1), we speculated that presynaptic mechanisms might be differentially involved in the expression of mGluR-LTD in the DH and VH sectors. To address this, we measured PPF before and after DHPG treatment in DH and VH slices, both across a range of inter-stimulus intervals at fixed time points (30 min before and 1 h after DHPG) and throughout the experimental time course with a 100 ms inter-stimulus interval (Figure 3). In line with previous studies, we found DHPG-LTD to be accompanied by an increase in PPF in both DH and VH slices (Figure 3(a)–(d)). However, the percentage change in PPF across the range of inter-stimulus intervals was significantly larger in VH compared to DH slices (Figure 3(e); two-way ANOVA: *F*_(1,40)_ = 21.74, *p* < 0.01), suggesting that presynaptic mechanisms may play a greater role in the expression of mGluR-LTD in the VH.

### DHPG-induced plasticity of intrinsic neuronal excitability is larger in the VH

In addition to the induction of LTD, activation of group I mGluRs has been shown to induce various forms of intrinsic plasticity leading to persistent changes in neuronal excitability. For example, a brief application of DHPG has been reported to result in a depolarisation of the resting membrane potential, an increase in input resistance, and a depression of the spike afterhyperpolarisation, all of which persist after washout of the agonist (Brager and Johnston, 2007; Gereau and Conn, 1995; Ireland et al., 2004). Although these forms of intrinsic plasticity are generally considered to lead to increased neuronal excitability (Ireland and Abraham, 2002), their overall effect on neuronal I/O function is unclear, with one study reporting a long-lasting depression of action potential generation in area CA1 after DHPG treatment (Clement et al., 2009). We have therefore investigated the effect of group I mGluR activation on neuronal I/O function by examining E-S coupling in the hippocampal CA1 region, and we have considered whether group I mGluR-mediated plasticity of intrinsic neuronal excitability differs in the DH and VH sectors. We did this in the same set of experiments in which we examined LTD of the fEPSP (shown in Figure 2(a)) by simultaneously recording the somatic PS via a second electrode placed in the stratum pyramidale and comparing E-S curves generated before and 40 min after DHPG treatment (100 µM, 10 min).

**Figure 4.**
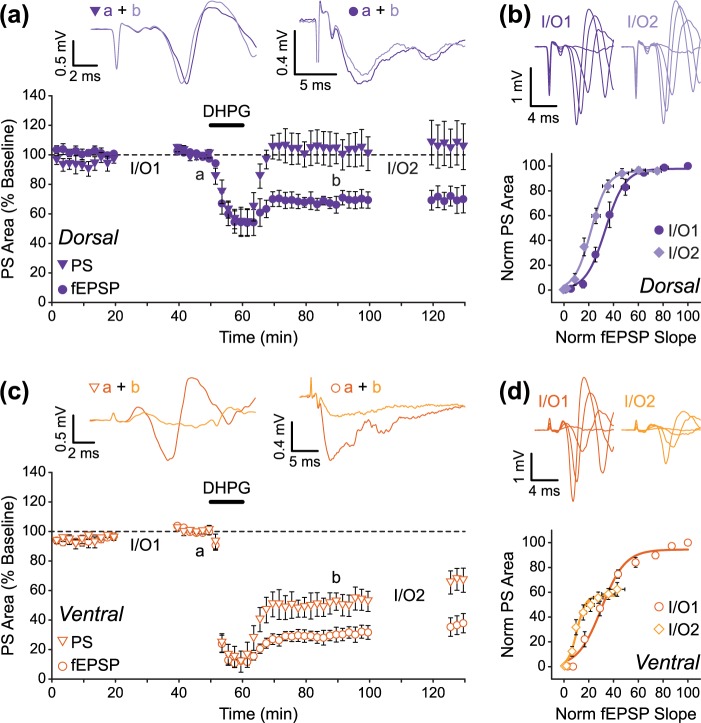
DHPG-induced E-S potentiation is larger in ventral compared to dorsal hippocampal slices. (a and b) Data from DH (*n* = 5) showing DHPG produces a long-lasting potentiation of E-S coupling. Although the fEPSP remains depressed following washout of DHPG, the PS quickly recovers to baseline levels (a). This produces a leftward shift in the E-S curve (b). (c and d) Although E-S potentiation is larger in VH slices (*n* = 5), there is also a reduction in maximal PS that can be evoked by stimulation (d). Data are from the same experiments shown in Figure 2 (fEPSP data in panels (a) and (c) are replotted from Figure 2(a)).

In DH slices, the PS was only transiently depressed by DHPG application, and it rapidly returned to baseline levels upon DHPG washout (Figure 4(a)). Since the fEPSP remained depressed in these experiments, this suggested that a long-lasting potentiation of E-S coupling had occurred following DHPG treatment. Indeed, the induction of E-S potentiation by DHPG was confirmed by a leftward shift in the E-S curves (Figure 4(b)), such that a significantly smaller fEPSP was required to elicit a half-maximal PS after DHPG (−36% ± 5% change in E_50_, *n* = 5; paired *t*-test: *t*_(4)_ = 8.29, *p* < 0.01). In VH slices, although the PS stayed depressed after washout of DHPG, the level of sustained depression was less than that of the simultaneously recorded fEPSP (Figure 4(c)), and DHPG-induced E-S potentiation was again evident as a leftward shift in the E-S curves (Figure 4(d); −63% ± 7% change in E_50_, *n* = 5; paired *t*-test: *t*_(4)_ = 5.44, *p* < 0.01). Moreover, we found that the magnitude of E-S potentiation induced by DHPG in these VH slices was significantly larger than the E-S potentiation induced in DH slices (unpaired *t*-test (DH vs VH): *t*_(8)_ = 3.05, *p* < 0.05). Interestingly, the E-S curves generated in VH slices also revealed a second long-lasting effect of DHPG on the PS recorded in the stratum pyramidale. We found that, 40 min after DHPG treatment, the maximal PS that could be elicited in response to Schaffer collateral stimulation was significantly depressed relative to control (pre-DHPG) levels (Figure 4(d); −39% ± 5% change in maximal PS, *n* = 5; paired *t*-test: *t*_(4)_ = 7.06, *p* < 0.01). This was in contrast to our experiments using DH slices, in which this DHPG-induced depression of the maximal PS was not apparent (Figure 4(b); −3% ± 3% change in maximal PS, *n* = 5; paired *t*-test: *t*_(4)_ = 1.12, *p* > 0.05).

These results therefore show that activation of group I mGluRs by DHPG has two distinct effects on neuronal I/O function in the hippocampal CA1 region: (1) a long-lasting increase in the likelihood of action potential firing in response to excitatory synaptic input (E-S potentiation) and (2) a persistent depression of the maximal PS, which presumably reflects a reduction in the total number of cells that are able to contribute action potentials to the evoked population response recorded in the stratum pyramidale. We also show that, like DHPG-LTD of synaptic transmission, these forms of group I mGluR-mediated intrinsic plasticity are more prominently expressed in the VH compared to the DH.

### The VH has a greater ability to exhibit group I mGluR-mediated plasticity

In the experiments described above, we used 100 µM of the racemic (*RS*) form of DHPG to selectively activate group I mGluRs. This is the concentration that is commonly used to induce LTD in slice studies and is near maximal at activating group I mGluRs in native and recombinant systems (Ito et al., 1992; Schoepp et al., 1994). However, full concentration–response curves for DHPG-LTD in hippocampal slices have not previously been reported, so the maximal level of LTD that can be induced by DHPG is unknown. Furthermore, we were interested to know if the differential effects of 100 µM DHPG in our experiments with DH and VH slices were primarily due to a difference in the ability of the DH and VH to exhibit group I mGluR-dependent plasticity (i.e. if the VH is capable of a greater tissue response), or if DHPG was somehow acting with a greater potency in VH slices compared to DH slices.

**Figure 5.**
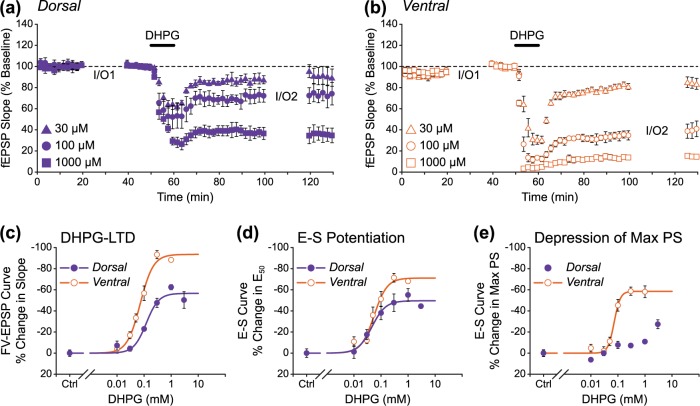
Ventral hippocampus is more sensitive than dorsal hippocampus to the actions of DHPG. (a and b) Pooled data showing that the concentration-dependent reduction in fEPSP slope is smaller for (a) DH slices than for (b) VH. Three concentrations of DHPG are shown here. (c–e) Concentration–response curves for the effect of DHPG on input/output relationships. The following concentrations of DHPG were used: Ctrl (no drug), 10 µM, 30 µM, 50 µM (VH only), 100 µM, 300 µM, 1 mM, and 3 mM (DH only). For all concentrations, *n* = 4. For all measurements, the maximal effect is larger in VH than in DH. Note that it is not possible to fit a curve to the dorsal data in (e).

To address this, we carried out concentration–response curves for DHPG-induced synaptic and intrinsic plasticity in both DH and VH slices. As before, I/O curves generated before and 40 min after DHPG (10 min applications) were compared to assess the levels of long-term plasticity (*n* = 4 for each concentration of DHPG; Figure 5). Interestingly, we found that 100 µM DHPG induced submaximal levels of synaptic LTD (Figure 5(a)–(c)) and E-S potentiation (Figure 5(d)) in both DH and VH slices, indicating that this concentration of DHPG may not fully activate group I mGluRs in adult rat hippocampal slices. Furthermore, we found that the maximal levels of both synaptic LTD and E-S potentiation that could be induced by DHPG differed significantly in DH and VH slices, with maximal LTD measured as −57% ± 5% versus −94% ± 4% (Figure 5(c); extra sum-of-squares *F* test: *F*_(1,50)_ = 15.31, *p* < 0.01) and maximal E-S potentiation measured as −50% ± 4% versus −71% ± 4% (Figure 5(d); extra sum-of-squares *F* test: *F*_(1,50)_ = 12.65, *p* < 0.01) in DH versus VH slices, respectively. Similarly, DHPG was more effective at inducing a persistent depression of the maximal PS in VH slices (−59% ± 3% maximal effect), and this form of plasticity was largely absent in DH slices apart from at the highest concentration of DHPG used (−27% ± 8% change in maximal PS with 3 mM DHPG; Figure 5(e)).

These results therefore suggest that the different magnitudes of synaptic and intrinsic plasticity induced by 100 µM DHPG in DH and VH slices are primarily due to a difference in the ability of the DH and VH sectors to exhibit group I mGluR-dependent forms of plasticity, with the VH capable of a greater tissue response. However, we also found that there is a small (~1.7-fold) but significant difference in the potency of DHPG with respect to the induction of synaptic LTD in the two slice preparations, with DHPG acting more potently in VH slices than in DH slices (Figure 5(c); DH EC_50_ = 120 µM, VH EC_50_ = 72 µM; extra sum-of-squares *F* test: *F*_(1,50)_ = 4.50, *p* < 0.05). In contrast, the potency of DHPG with respect to the induction of E-S potentiation was not significantly different in DH and VH slices (Figure 5(d); DH EC_50_ = 46 µM, VH EC_50_ = 55 µM; extra sum-of-squares *F* test: *F*_(1,50)_ = 0.40, *p* > 0.05).

## Discussion

Since its first description in the mid-to-late 1990s (Kemp and Bashir, 1999; Overstreet et al., 1997; Palmer et al., 1997), group I mGluR-dependent LTD has been extensively studied. Despite these nearly two decades of research, however, disagreement remains over the precise mechanisms involved in the induction and expression of mGluR-LTD, with conflicting reports in the published literature. For example, some studies have suggested that activation of only mGlu5 is required for the induction of mGluR-LTD (Huang et al., 2004; Moult et al., 2008), whereas other studies have found a role for both mGlu1 and mGlu5 (Volk et al., 2006). Contrasting results have also been reported with respect to the involvement of a variety of kinases in mGluR-LTD, including p38 MAPK, ERK, PI3K, PKC, and CaMKII (Gallagher et al., 2004; Hou and Klann, 2004; Mockett et al., 2011; Moult et al., 2008; Oliet et al., 1997; Schnabel et al., 1999a, 1999b), and the requirement for protein synthesis has also been subject to controversy (Huber et al., 2000; Moult et al., 2008). Furthermore, whether mGluR-LTD is expressed presynaptically (Fitzjohn et al., 2001; Qian and Noebels, 2006; Tan et al., 2003; Watabe et al., 2002) or postsynaptically (Moult et al., 2006; Waung et al., 2008; Xiao et al., 2001; Zhang et al., 2008) is still a matter of debate. Although the reasons for these discrepancies are unknown, it seems likely that methodological differences between research groups could account for at least some of them. For example, the molecular and synaptic mechanisms underlying mGluR-LTD have been shown to vary depending on the age of animals used to prepare slices (Kumar and Foster, 2007; Nosyreva and Huber, 2005), as well as the protocol used for induction (Connelly et al., 2011). Our finding that the magnitude of DHPG-LTD differs in DH and VH slices now raises the intriguing possibility that the mechanisms involved in mGluR-LTD might differ in the DH and VH sectors. Indeed, various slice preparation methods, which are likely to yield slices from distinct locations along the hippocampal longitudinal axis, have previously been used in mGluR-LTD studies, including parasagittal (Moult et al., 2006), horizontal (Rohde et al., 2009), coronal (Ronesi and Huber, 2008), and transverse (Faas et al., 2002). As such, the use of different hippocampal slice preparations by different research groups could account for some of the conflicting results that have previously been reported. In particular, our results showing that a change in PPF after DHPG treatment is larger in VH compared to DH slices suggests that presynaptic expression mechanisms might play a greater role in mGluR-LTD in the VH.

Alongside the first descriptions of mGluR-LTD in the hippocampus, there were reports that pharmacological activation of group I mGluRs could also elicit inward currents and modulate the intrinsic properties of individual hippocampal neurons (Congar et al., 1997; Davies et al., 1995). In some studies, these effects were found to persist long after washout of the agonist, suggesting that group I mGluR activation can result in a long-lasting enhancement of intrinsic neuronal excitability (Ireland and Abraham, 2002). Enhanced excitability induced by group I mGluR activation has also been reported at the network level, with the observation that DHPG application leads to a persistent prolongation of spontaneous epileptiform bursting activity in disinhibited hippocampal slices (Merlin and Wong, 1997; Piccinin et al., 2008). Consistent with these previous reports, we found that DHPG induced a long-lasting enhancement of neuronal I/O function when it was applied under conditions in which inhibitory synaptic transmission was blocked by picrotoxin, suggesting that this form of E-S potentiation is expressed via an intrinsic mechanism (an increase in the excitability of the CA1 pyramidal cells) rather than via a synaptic mechanism (a shift in the balance of excitatory and inhibitory synaptic drive). Interestingly, although E-S potentiation induced by pharmacological activation of group I mGluRs in the hippocampus has been reported in several previous studies, the effect was either found to be short-lasting (Cohen et al., 1999), or was attributed to reduced inhibitory drive rather than a change in intrinsic neuronal excitability (Breakwell et al., 1996; Zhang and Alger, 2010). The DHPG-induced E-S potentiation we describe in this study therefore appears to be a hitherto unreported form of mGluR-mediated E-S plasticity that has an intrinsic rather than synaptic mechanism of expression, is long-lasting, and like DHPG-LTD is expressed more prominently in the VH.

An unexpected finding of this study was that activation of group I mGluRs by 100 µM DHPG also induces a large and persistent depression of the maximal PS in VH slices. Although it is possible that a modulation of individual action potential waveforms might underlie this effect (Carlier et al., 2006), a persistent reduction in the number of cells contributing action potentials to the evoked population response is a more likely candidate mechanism given the large (~40%) depression of the PS area we observed in these experiments. The fact that DHPG treatment can simultaneously induce a potentiation of E-S coupling and a depression of the PS is initially somewhat curious, since E-S potentiation is indicative of enhanced excitability (an increase in the likelihood of action potential firing in response to excitatory input) whereas a depression of the PS would appear to reflect a reduced excitability (a decrease in action potential firing across the neuronal population overall). However, these seemingly opposing effects of DHPG might be explained if the depression of the maximal PS is in fact a manifestation of an underlying enhancement of neuronal excitability mediated by group I mGluR activation rather than representing a true depression of excitability. For example, enhanced excitability could lead to a reduction in the number of CA1 pyramids able to contribute to the PS during an evoked event via a depolarisation block of action potential generation (Bianchi et al., 2012) or via an increase in the proportion of cells that are in a refractory state due to increased spontaneous firing (Chuang et al., 2002). Interestingly, the DHPG-induced depression of the maximal PS we report in this study might explain why, under certain conditions, DHPG treatment can lead to an apparent reduction, rather than enhancement, of neuronal excitability (Clement et al., 2009).

The principal finding of this study is that the VH has a greater ability to exhibit group I mGluR-dependent forms of long-term plasticity when compared to the DH. Since the precise mechanisms underlying group I mGluR-dependent forms of plasticity have not yet been fully elucidated, the possible reasons for this dorsal/ventral difference are not immediately clear. One possibility is that there is a differential expression of group I mGluRs along the hippocampal longitudinal axis, with a higher receptor density in the VH compared to the DH. Indeed, our finding that DHPG induces both synaptic and intrinsic forms of plasticity with greater efficacy in VH slices is consistent with this hypothesis, since increases in receptor density have been shown to lead to higher agonist efficacies in recombinant expression systems (Gazi et al., 1999; Hermans et al., 1999), and increased expression of mGlu5 has previously been associated with an enhanced level of DHPG-LTD in a rat model of treatment-resistant depression (Pignatelli et al., 2013). Alternatively, the higher efficacy of DHPG in VH slices might reflect a stronger coupling of group I mGluRs to effector systems in the VH compared to the DH. Interestingly, we found that DHPG acted not only with a higher efficacy but also with a greater potency in VH slices with respect to the induction of synaptic LTD. This result is intriguing as it raises the possibility that two or more distinct mechanisms may contribute differentially to the induction or expression of mGluR-LTD in the DH and VH sectors. For example, if both mGlu1 and mGlu5 play a role in LTD induction (Volk et al., 2006), then different relative expression levels of these two receptor subtypes in the DH versus the VH might explain the observed difference in potency, since DHPG is thought to act more potently at mGlu5 than at mGlu1 (Conn and Pin, 1997). Alternatively, the greater potency of DHPG in the VH could reflect a differential activation of downstream signalling pathways or a differential involvement of presynaptic and postsynaptic expression mechanisms. One further possibility to explain the larger magnitude of group I mGluR-dependent plasticity in the VH is that the ability of a particular brain region to exhibit certain forms of plasticity might depend to some extent on the basal conditions or intrinsic properties of neurons and synapses present. Indeed, DH and VH CA1 pyramidal neurons are known to be different in their intrinsic excitability (Dougherty et al., 2012), and we have shown here that basal synaptic efficacy and E-S coupling also differ between these hippocampal regions. As such, it is possible that the ability to exhibit a large synaptic LTD might depend, for example, on conditions in which basal synaptic transmission and release probability are initially high. Similarly, a large magnitude of E-S potentiation may require an initially low level of E-S coupling in order to be expressed. Although speculative, this idea is consistent with the findings we present in this study and also with previous reports showing that the magnitude of LTP is larger in the DH, where release probability is low, compared to the VH, where release probability is high (Papatheodoropoulos and Kostopoulos, 2000a, 2000b).

In conclusion, we have shown here that synaptic and intrinsic forms of long-term plasticity induced by activation of group I mGluRs differ in DH and VH slices. This differential ability of the DH and VH sectors to exhibit group I mGluR-mediated plasticity may help to explain some of the conflicting results that have previously been reported with respect to the mechanisms underlying mGluR-LTD. Furthermore, we report that basal levels of excitatory synaptic transmission and E-S coupling are different in DH and VH slices. Taken together, these findings lend further support to the idea that, in addition to differences in extrinsic connectivity, differences in the information processing properties of local hippocampal circuits may also contribute to the functional differentiation of the hippocampal longitudinal axis.
